# The Monocyte-to-Lymphocyte Ratio Predicts Acute Kidney Injury After Acute Hemorrhagic Stroke

**DOI:** 10.3389/fneur.2022.904249

**Published:** 2022-06-20

**Authors:** Fen Jiang, Jialing Liu, Xin Yu, Rui Li, Run Zhou, Jianke Ren, Xiangyang Liu, Saili Zhao, Bo Yang

**Affiliations:** ^1^Department of Nephrology, The First Affiliated Hospital, Hengyang Medical School, University of South China, Hengyang, China; ^2^Department of Gastroenterology, The First Affiliated Hospital, Hengyang Medical School, University of South China, Hengyang, China; ^3^Department of Clinical Medicine, Xiangnan University, Chenzhou, China; ^4^Department of Nursing, Hengyang Medical School, The First Affiliated Hospital, University of South China, Hengyang, China

**Keywords:** monocyte-to-lymphocyte ratio, acute kidney injury, acute hemorrhagic stroke, prediction, in-hospital mortality

## Abstract

**Objectives:**

Acute kidney injury (AKI) is a serious complication of acute hemorrhagic stroke (AHS). Early detection and early treatment are crucial for patients with AKI. We conducted a study to analyze the role of the monocyte-to-lymphocyte ratio (MLR) in predicting the development of AKI after AHS.

**Methods:**

This retrospective observational study enrolled all subjects with AHS who attended the neurosurgical intensive care unit (NSICU) at the First Affiliated University of South China between 2018 and 2021. Patient demographics, laboratory data, treatment details, and clinical outcomes were recorded.

**Results:**

Of the 771 enrolled patients, 180 (23.3%) patients developed AKI. Compared to patients without AKI, those with AKI had a higher MLR and the neutrophil-lymphocyte ratio (NLR) at admission (*P* < 0.001). The MLR and the NLR at admission were associated with an increased AKI risk, with odds ratios (ORs) of 8.27 (95% CI: 4.23, 16.17, *p* < 0.001) and 1.17 (95% CI: 1.12, 1.22, *p* < 0.001), respectively. The receiver operating characteristic curve (ROC) analysis was conducted to analyze the ability of the MLR and NLR to predict AKI, and the areas under the curve (AUCs) of the MLR and the NLR were 0.73 (95% CI: 0.69, 0.77, *p* < 0.001) and 0.67 (95% CI: 0.62, 0.72, *p* < 0.001), with optimal cutoff values of 0.5556 and 11.65, respectively. The MLR and the NLR at admission were associated with an increased in-hospital mortality risk, with ORs of 3.13 (95% CI: 1.08, 9.04) and 1.07 (95% CI: 1.00, 1.14), respectively. The AUCs of the MLR and the NLR for predicting in-hospital mortality were 0.62 (95% CI: 0.54, 0.71, *p* = 0.004) and 0.52 (95% CI: 0.43, 0.62, *p* = 0.568), respectively. The optimal cutoff value for the MLR was 0.7059, with a sensitivity of 51% and a specificity of 73.3%.

**Conclusions:**

MLR and NLR measurements in patients with AHS at admission could be valuable tools for identifying patients at high risk of early AKI. The MLR was positively associated with in-hospital mortality and the NLR showed a weak ability for the prediction of in-hospital mortality.

## Introduction

Acute stroke is a common and serious complication that increases mortality and severe disability worldwide and imposes a substantial socioeconomic burden (1, 2). It has been reported that the prevalence rate of survival after stroke in the elderly population is 4.94% in China (3). Stroke can be classified into two main types: ischemic stroke, which accounts for 85% of all acute strokes, and acute hemorrhagic stroke (AHS), which accounts for 15% of all acute hemorrhagic strokes and has a high mortality rate (4). Stroke is often associated with multiple complications, such as infection, malnutrition, and thrombosis (5). Acute kidney injury (AKI) refers to a rapid decline in renal function within hours to days, which relates to high mortality and affects prognosis (6). An increasing number of studies on AKI after stroke have been conducted recently, and it has been reported that the incidence of AKI ranges from 6.8 to 26.7% after acute hemorrhagic stroke (7, 8). Previous studies have suggested that AKI increases the risk of in-hospital mortality and severe disability following stroke (7, 9, 10). Therefore, the early identification of AKI has become a focus in the clinical setting. Currently, the diagnosis of AKI is still based on the changes in urine volume and serum creatine (sCr), which are recognized as insensitive in early diagnosis (11). In recent decades, many specialists have attempted to identify ideal biomarkers to predict AKI, and an increasing number of novel biomarkers, such as kidney injury molecular-1 and cystatin C, have been investigated for their value in early AKI detection (12–14). However, for some reason, they have not yet been widely applied in clinical practice. Inflammatory mediators are involved in the onset and progression of AKI (15). Patients with AKI present with changes in the morphology and the function of vascular endothelial cells (16, 17). The monocyte-to-lymphocyte ratio (MLR) and the neutrophil-to-lymphocyte ratio (NLR) are reliable inflammatory biomarkers that are calculated from complete blood counts (18, 19). Previous studies have demonstrated that the NLR is associated with hospital mortality in patients diagnosed with acute stroke (19, 20). Ultimately, the relationship between the MLR and the NLR in AKI is still unclear. Therefore, we sought to explore the association of the MLR and the NLR with AKI in patients diagnosed with AHS.

## Methods

### Study Design

We extracted the data of 929 subjects diagnosed with AHS who attended the neurosurgical intensive care unit (NSICU) of the First Affiliated University of South China from July 2018 to August 2021 in the hospital medical record. The First Affiliated University of South China is a teaching hospital with 2,300 beds, and there are 23 beds in the NSICU. In this study, the population comprised patients older than 18 years. Patients were excluded if they had AKI before admission; were admitted to the NSICU for <24 h; had preexisting chronic renal dysfunction requiring renal replacement therapy (RRT); had a tumor or rheumatism; had undergone kidney transplantation; had a second AHS attack; or had missing routine blood test or renal function data within 7 days after admission to the NSICU.

### Data Collection

The following variables were collected: sex, age, preexisting clinical conditions, inflammatory markers, blood biochemistry, complete blood count, and Glasgow coma scale (GCS) score. The MLR and the NLR were defined as the ratios of the monocyte count and neutrophil count to the lymphocyte count, respectively, such counts were calculated from a peripheral blood sample on admission by fluorescent flow cytometry (18, 20). AHS was defined as the onset of symptoms and the evidence on cranial CT images. AKI was defined as the rapid decrease of kidney function within a few hours or days. AKI was diagnosed with the Kidney Disease: Improving Global Outcomes (KDIGO) criteria as follows: (a) a rise in sCr of ≥0.3 mg/dL (26.5 μmol/L) within 48 h; (b) an increase in sCr to ≥1.5 times in the past 7 days; or (c) a urine volume of ≤ 0.5 ml/kg/h for 6 h (21). Patients with sCr increase 1.5 to 1.9 times baseline or increase to ≥0.3 mg/dl (≥26.5 μmol/l) or with urine output <0.5 ml/kg/hour for 6 to 12 h are classified as being at stage 1; patients with sCr increased 2.0 to 2.9 times baseline or increase to <0.5 ml/kg/hour for ≥12 h are classified as being at stage 2; stage 3 was marked for patients with Scr increased 3.0 times baseline or increase to ≥4.0 mg/dl (≥353.6 μmol/l) or initiation of renal replacement therapy, or in patients <18 years, a decrease in estimated baseline glomerular filtration (eGFR) to <35 ml/min per 1.73 m^−2^, <0.3 ml/kg/hour for ≥24 h or anuria for ≥12 h (21). The lowest value of sCr measured in the general ward or emergency clinic before attending the NSICU was taken as the baseline creatinine value. When the value was missing, the sCr level was calculated using the modifications of the diet in the renal disease method, assuming a normal glomerular filtration rate of 75 ml·min^−1^·1.73 m^−2^ (22). The eGFR was calculated according to the Chronic Kidney Disease Epidemiology Collaboration equation (22).

### Statistical Analysis

SPSS 16 software was used to analyze all data (Chicago, IL, USA). Continuous data are presented as medians with interquartile ranges, while categorical variables are shown as frequency counts (percent). The Chi-square tests were used to compare categorical variables between groups. Comparisons between continuous variables were made using *t*-tests. Spearman's correlation was applied to analyze the association of the MLR with other variables. The relationships of the MLR and the NLR with AKI and prognosis were subjected to multivariable logistic regression, and the results are given as odds ratios (ORs). The model of AKI was adjusted for age, sex, eGFR, baseline Scr, blood urea nitrogen, diabetes mellitus, coronary artery disease, hypertension, the Glasgow coma scale (GCS) score, use of angiotensin-converting enzyme inhibitors (ACEI), angiotensin receptor blockers (ARB), and contrast agents. The model of hospital mortality was adjusted for sex (men), age, hypertension, coronary artery disease, diabetes mellitus and hemoglobin, triglyceride, cholesterol, albumin, and the GCS score. The receiver operating characteristic (ROC) curves were applied to evaluate the predictive usefulness of the MLR and the NLR for the development of AKI and in-hospital mortality. The Youden index was used to calculate cutoff values as well as the sensitivity and specificity of the parameters. For the study, a two-tailed *p* < 0.05 indicated statistical significance.

## Results

### Patient Characteristics

**Figure 1:**
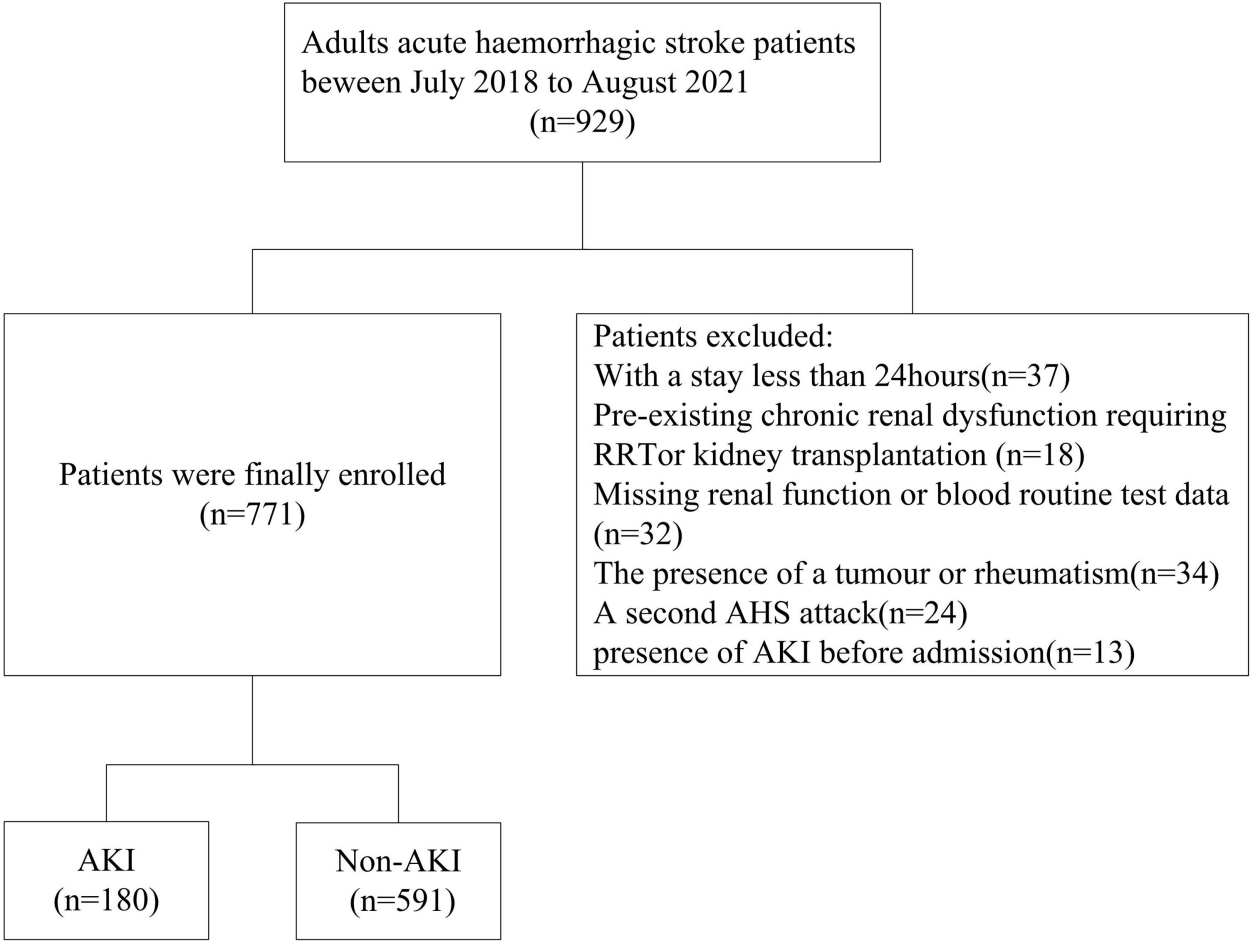
Flowchart for patient selection. AKI, acute kidney injury.

Of the 929 patients diagnosed with AHS who attended the NSICU during the screening period, 771 patients were ultimately included (Figure 1). A total of 497 (64.5%) men and 274 (35.5%) women were recruited. Of the 180 (23.3%) subjects who developed AKI, 125 (16.2%) had stage 1 AKI, 25 (3.2%) had stage 2 AKI, and 30 (3.9%) had stage 3 AKI. The average age of the patients was 60.53 ± 11.84 years. Male sex, patients with diabetes mellitus or coronary artery disease, and those with a lower eGFR or higher baseline sCr and blood urea nitrogen (BUN) levels had a higher likelihood of developing AKI than their counterparts (*p* < 0.05). Remarkable differences were observed in triglyceride, procalcitonin (PCT), and C-reactive protein (CRP) levels and the GCS scores between the two groups (*p* < 0.05). Patients with AKI had a higher MLR and NLR at admission. Overall, there were 88(11.4%) patients using ACEI, 229 (29.7%) patients using ARB, and 562 (72.9%) patients using contrast agents during the hospital stay. Also, 49 (6.4%) patients died in the hospital and 9 (0.9%) received renal replacement treatment, but the number of patients with assisted ventilation was as high as 412 (53.4%). Moreover, AKI patients had higher rates of mortality, renal replacement therapy (RRT), and assisted ventilation, but no noticeable difference was observed in the length of hospital stay. Compared to the non-AKI groups, the AKI group did not have a higher rate of using contrast agents, ARB, or ACEI (Table 1).

**Table 1:** Baseline characteristics and outcomes of the patients.

**Variables**	**ALL**	**AKI**	**Non-AKI**	***p* value**
	**(*n* = 771)**	**(*n* = 180)**	**(*n* = 591)**	
Sex, male (%)	497 (64.5%)	143 (79.4%)	37 (6.3%)	<0.001^*^
Age (years)	60.53 ± 11.84	61.52 ± 11.36	60.23 ± 11.98	0.200
Primary disease				
Cerebrovascular malformation	144 (18.7%)	35 (19.44%)	109 (18.44%)	0.763
Hypertension	645 (83.7%)	155 (86.1%)	25 (4.2%)	0.310
Diabetes mellitus	91 (11.8%)	39 (21.7%)	52 (8.8%)	<0.001^*^
Coronary artery disease	35 (4.5%)	15 (8.3%)	20 (3.4%)	0.005
eGFR (ml/min/1.73 m^2^)	84.13 ± 25.28	74.94 ± 30.54	86.93 ± 22.74	<0.001^*^
Laboratory index at NSICU admission				
Baseline sCr (umol/L)	87.09 ± 33.71	104.76 ± 47.29	81.71 ± 26.08	<0.001^*^
BUN (mmol/L)	5.59 ± 2.75	6.72 ± 3.61	5.24 ± 4.42	<0.001^*^
Albumin (g/L)	42.8 ± 4.2	42.49 ± 4.42	42.89 ± 4.13	0.271
Triglyceride (mmol/L)	1.64 (0.81, 1.86)	1.78 ± 1.08	1.18 (0.81, 1.86)	<0.001^*^
Cholesterol (mmol/L)	4.27 ± 0.97	4.36 ± 0.97	4.25 ± 0.97	0.201
PCT (ng/mL)	0.56 (0.04, 0.75)	1.24 (0.80, 0.72)	0.09 (0.043, 0.21)	0.003^*^
White blood count (mm^3^)	11.58 ± 4.24	12.12 ± 4.78	11.41 ± 4.47	0.047^*^
CRP (mg/L)	24.43 ± 4.89	25.78 (17.02, 131.59)	3.60 (0.73, 22.36)	<0.001^*^
Potassium (mmol/L)	3.67 ± 0.49	3.69 ± 0.57	3.67 ± 0.46	0.545
Hemoglobin (g/L)	133.59 ± 47.19	138.06 ± 92.52	132.23 ± 13.36	0.147
GCS score	10.27 ± 3.58	10.64 ± 3.47	9.07 ± 3.67	<0.001^*^
PLR	224.61 ± 165.53	223.56 ± 131.75	224.93 ± 174.63	0.923
MLR	0.5374 ± 0.3001	0.7584 ± 0.2610	0.4701 ± 0.2765	<0.001^*^
NLR	9.10 ± 4.76	11.67 ± 5.67	8.31 ± 4.02	<0.001^*^
Medication during admission				
ACEI	88 (11.4%)	19 (10.56%)	69 (11.67%)	0.674
ARB	229 (29.7%)	58 (32.2%)	171 (28.9%)	0.398
Contrast agent	562 (72.9%)	13 (72.2%)	432 (73.1%)	0.817
Outcome				
Hospital mortality	49 (6.4%)	27 (15%)	22 (3.7%)	<0.001^*^
Renal replacement treatment	9 (0.9%)	8 (4.4%)	1 (0.16%)	<0.001^*^
Ventilation	412 (53.4%)	132 (73.3%)	280 (47.38%)	<0.001^*^
Hospital stay	23.87 ± 18.43	22.34 ± 19.57	24.33 ± 18.06	0.203^*^

eGFR, estimate baseline glomerular filtration rate; BUN, blood urea nitrogen; sCr, serum creatinine; PCT, procalcitonin; MLR, monocyte-to-lymphocyte ratio; CRP, C-reactive protein; NLR, neutrophil-to-lymphocyte ratio; PLR, platelet-to-lymphocyte ratio; ARB, angiotensin- receptor blocker; GCS, Glasgow coma score; ACEI, angiotensin-converting enzyme inhibitor. ^*^p < 0.05.

### The Relationship of the MLR With Other Variables at Baseline

Male sex, baseline sCr, BUN, CRP, and PCT levels, the NLR, the GCS scores, and the PLR displayed a moderate correlation with the MLR (*p* < 0.05), while age, albumin, use of ventilation, and triglyceride levels had no relationship with the MLR (*p* > 0.05) (Table 2).

**Table 2:** Associations with the baseline MLR and other factors.

**Variable**	**r**	***p*-value**
Age (years)	0.06	0.083
Sex (male)	0.15	<0.001^*^
Baseline sCr (mmol/L)	0.15	<0.001
BUN (mmol/L)	0.12	0.001^*^
Albumin (g/L)	0.03	0.921
Triglycerides (mmol/L)	0.03	0.451
CRP (mg/L)	0.18	<0.001^*^
PCT (ng/mL)	0.19	<0.001^*^
GCS score	0.14	<0.001^*^
NLR	0.35	<0.001^*^
PLR	0.25	<0.001^*^
Ventilation	0.06	0.077

MLR, monocyte-to-lymphocyte ratio; CRP, C-reactive protein; sCr, serum creatinine; BUN, blood urea nitrogen; PCT: procalcitonin; NLR, neutrophil-to-lymphocyte ratio; GCS, Glasgow coma score. PLR, platelet-to-lymphocyte ratio. ^*^p < 0.05.

### The MLR Predicts the Incidence of AKI

According to the multivariable logistic regression model, after adjustment for age, sex (male), baseline sCr level, diabetes mellitus, coronary artery disease, GCS score, and the use of contrast agents, ARB and ACEI, the MLR and NLR at admission were associated with an increased AKI risk, with ORs of 8.27 (95% CI: 4.23, 16.17, *p* < 0.001) and 1.17 (95% CI: 1.12, 1.2, *p* < 0.001), respectively. The ORs for AKI were 1.00 (95% CI: 0.99, 1.01, *p* = 0.251) for CRP and 1.04 (95% CI: 0.96, 1.01, *p* = 0.341) for PCT. The OR for AKI was 2.95 (95%CI: 1.92, 4.53, *p* < 0.001) for ventilation (Table 3).

**Table 3:** The value of the MLR and NLR for the prediction of AKI analyzed by the multivariable logistic regression analysis.

**Variable**	**Unadjusted**		After adjustment^*^	
	**OR(95% CI)**	***p* value**	**OR(95% CI)**	***p* value**
MLR	10.51 (5.70, 19.38)	<0.001	8.27 (4.23, 16.17)	<0.001^*^
NLR	1.15 (1.11, 1.19)	<0.001	1.17 (1.12, 1.22)	<0.001^*^
CRP (mg/L)	1.01 (1.00, 1.01)	0.016	1.00 (0.99,1.01)	0.251
PCT (ng/mL)	1.10 (1.00, 1.20)	0.04	1.04 (0.96, 1.11)	0.341
Ventilation	3.05 (2.12,4.41)	<0.001	2.95 (1.92,4.53)	<0.001^*^
Sex (male)	2.59 (1.38, 3.85)	<0.001		
GCS	0.88 (0.84, 0.93)	<0.001		
Age (years)	1.01 (0.99, 1.03)	0.09		
Hypertension	1.03 (0.66, 1.76)	0.908		
Diabetes mellitus	2.29 (1.38, 3.81)	0.001		
Coronary artery disease	2.49 (1.12, 5.49)	0.024		
eGFR (ml/min/1.73 m^2^)	1.02 (1.00, 1.03)	0.019		
BUN (mmol/L)	1.20 (1.13,1.28)	<0.001		
Baseline Scr (mmol/L)	1.02 (1.01,1.03)	<0.001		
ACEI	0.89 (0.52,1.53)	0.674		
ARB	1.17 (0.82,1.67)	0.398		
Contrast agents	0.96 (0.66,1.39)	0.817		

Adjusted for age, sex, eGFR, baseline Scr level, blood urea nitrogen level, diabetes mellitus, coronary artery disease, and hypertension, GCS score, and the use of ACEIs, ARBs, and contrast agents. MLR, monocyte-to-lymphocyte ratio; NLR, neutrophil-to-lymphocyte ratio; CRP, C-reactive protein; PCT, procalcitonin; OR, odds ratio; CI, confidence interval; ACEI, angiotensin-converting enzyme inhibitors; ARB, angiotensin-receptor blockers; ssCr, serum creatinine. ^*^p < 0.05.

### Ability of the MLR and NLR to Predict AKI

**Figure 2:**
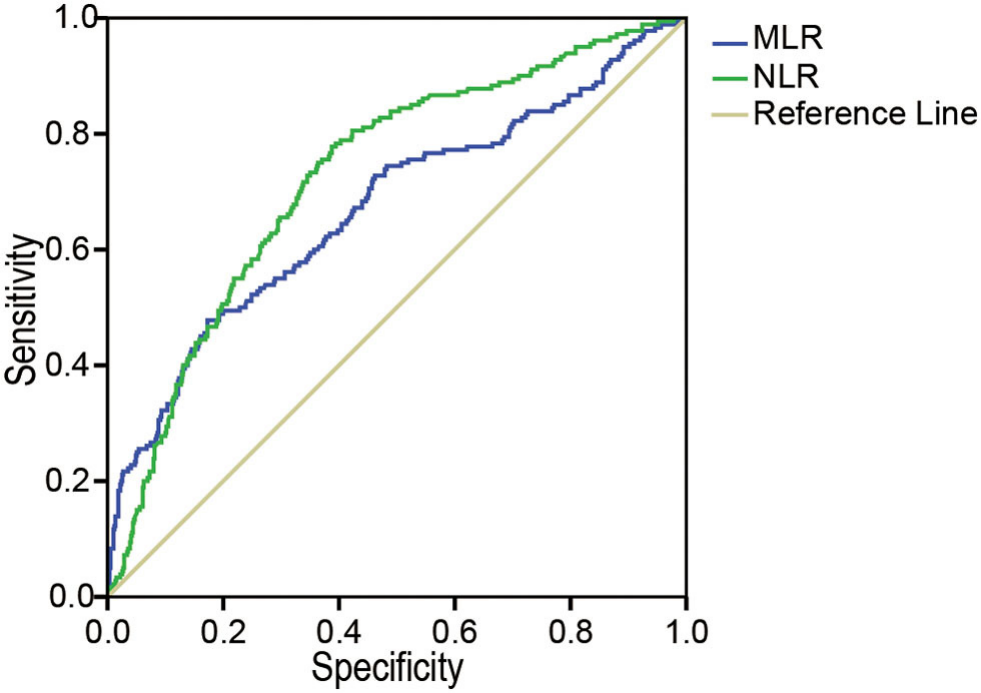
The ROC analysis of the MLR and the NLR for AKI. The area under the ROC of the MLR and NLR at admission to predict the development of AKI were 0.73 (95% CI: 0.69, 0.77, *p* < 0.001) and 0.67 (95% CI: 0.62, 0.72, *p* < 0.001), respectively. MLR, monocyte-to-lymphocyte ratio; NLR, neutrophil-to-lymphocyte ratio; AKI, acute kidney injury, ROC, receiver operating characteristics.

The area under the curve (AUC) for the ability of the MLR at admission to predict the development of AKI was 0.73 (95% CI: 0.69, 0.77, *p* < 0.001), with a cutoff value of 0.5556, a sensitivity of 77.8%, and a specificity of 61.3%. The AUC of the NLR for predicting AKI was 0.67 (95% CI: 0.62, 0.72, *p* < 0.001), with a cutoff value of 11.65, which best distinguished the occurrence of AKI; the sensitivity was 47.8%, and the specificity was 82.7% (Figure 2).

### The Association Between the MLR and In-hospital Mortality

To determine the value of the MLR and the NLR in predicting in-hospital mortality, a multivariable logistic regression was performed (Table 4). After adjusting for age, sex (male), diabetes mellitus, coronary artery disease, hypertension, hemoglobin, triglyceride, cholesterol, and albumin levels, and the GCS scores, the MLR and the NLR at admission were associated with increased in-hospital mortality risk, with ORs of 3.13 (95% CI, 1.08, 9.04) and 1.067 (95% CI, 1.00, 1.14), respectively. Meanwhile, the OR of AKI was 5.28 (95% CI 2.57, 10.84) for in-hospital mortality. No remarkable difference was observed between the CRP and PCT levels for in-hospital mortality.

**Table 4:** The association of the MLR and NLR with in-hospital mortality.

**Variable**	**Unadjusted**		**After adjustment**	
	**OR (95% CI)**	***p* value**	**OR (95% CI)**	***p* value**
AKI	2.17 (1.65, 2.85)	<0.001	5.28 (2.57, 10.84)	<0.001
MLR	3.38 (1.52, 7.53)	0.003	3.13 (1.08, 9.04)	0.035
NLR	1.04 (0.98, 1.10)	0.168	1.07 (1.00, 1.14)	0.043
PCT (ng/mL)	1.00 (0.90, 1.13)	0.920	1.00 (0.90, 1.11)	0.997
CRP (mg/L)	1.00 (0.99, 1.01)	0.399	1.01 (0.99, 1.01)	0.147
Age (years)	1.04 (1.01, 1.07)	0.003		
Sex (male)	1.57 (0.82, 3.01)	0.177		
Hypertension	0.86 (0.41, 1.82)	0.692		
Diabetes mellitus	3.35 (1.73, 6.50)	<0.001		
Coronary artery disease	1.41 (0.42, 4.77)	0.584		
Hemoglobin (g/L)	1.00 (0.99, 1.01)	0.849		
Triglycerides (mmol/L)	0.96 (0.78, 1.19)	0.719		
Cholesterol (mmol/L)	0.99 (0.72, 1.38)	0.993		
Albumin (g/L)	0.64 (0.34, 1.20)	0.164		
GCS	0.82 (0.75, 0.90)	<0.001		

Adjusted for sex (male), age, hypertension, coronary artery disease, diabetes mellitus and hemoglobin, triglyceride, cholesterol, and albumin levels, and GCS score. MLR, monocyte-to-lymphocyte ratio; NLR, neutrophil-to-lymphocyte ratio; CRP, C-reactive protein; PCT, procalcitonin; OR, odds ratio; CI, confidence interval; GCS, Glasgow coma score; AKI: acute kidney injury. *p < 0.05.

### The Prediction of In-hospital Mortality

**Figure 3:**
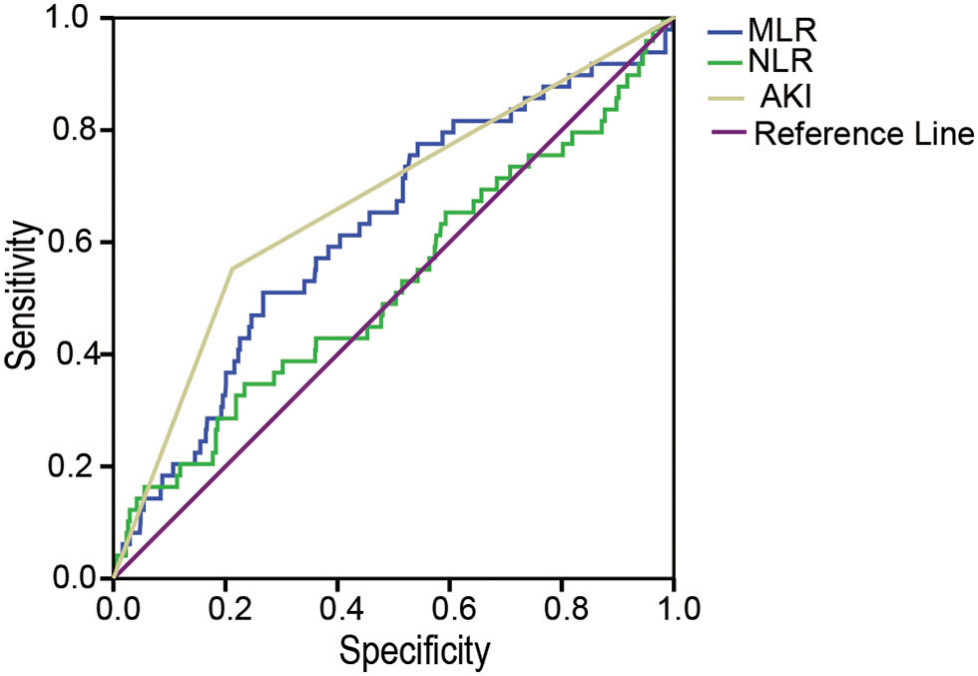
The ROC analysis of the MLR, the NLR, and AKI for in-hospital mortality. The area under the ROC of AKI, the MLR, and the NLR for predicting in-hospital mortality were 0.68 (95% CI: 0.60, 0.78, *p* < 0.001), 0.62 (95% CI: 0.54, 0.71, *p* = *0.0*04) and 0.52 (95% CI: 0.43, 0.62, *p* = 0.568), respectively. MLR, monocyte-to-lymphocyte ratio; NLR, neutrophil-to-lymphocyte ratio; AKI, acute kidney injury; ROC, receiver operating characteristics.

The AUCs of AKI, the MLR, and the NLR for predicting in-hospital mortality were 0.68 (95% CI: 0.60, 0.78, *p* < 0.001), 0.62 (95% CI: 0.54, 0.71, *p* = *0.0*04), and 0.52 (95% CI: 0.43, 0.62, *p* = 0.568), respectively. The optimal cutoff value for the MLR was 0.7059, with a sensitivity of 51% and a specificity of 73.3% (Figure 3).

## Discussion

In this single-center retrospective study of patients diagnosed with AHS, we assessed the role of the MLR and the NLR at admission in the prediction of the development of AKI and found that a higher MLR and NLR were associated with an increased risk of AKI; the MLR was superior to the NLR in predicting AKI. Meanwhile, the MLR and AKI showed the ability to predict in-hospital mortality.

The incidence of AKI in our study was 23.3%, but in a meta-analysis conducted by Zorrilla-Vaca et al. (7), the pooled prevalence rate of AKI after AHS was 19.0%. The higher incidence rate of AKI may be explained by the study population, which included patients admitted in NSICU. In our study, patients with AKI were predominantly male patients who had a lower eGFR and had diabetes mellitus or coronary artery disease. In contrast to previous studies, our results showed that PCT and CRP levels were not independent predictors of AKI (23, 24). However, unlike the results of previous studies, hypertension did not increase the risk of AKI progression in the current study (8).

To our knowledge, this study is the first to highlight the role of the MLR in the prediction of AKI in patients diagnosed with AHS. The results of this study provide evidence of the MLR as an independent predictive biomarker of AKI. A higher MLR was related to a 3.65-fold increased risk for AKI, and the AUC of the MLR was 0.73. This could be demonstrated by the idea that both immunological changes and inflammation lead to AKI (15, 17). In hemorrhagic stroke, brain tissue injuries, and internal injuries cause blood vessels to rupture, causing an abnormal accumulation of blood within the brain and leading to the activation of monocytes that induced more severe brain cell death and cerebral tissue damage. Increased monocytes and neutrophils are responsible for the higher levels of the MLR and the NLR. Neuroinflammation, blood–brain barrier dysfunction, and the interruption of blood flow are the main mechanisms (2, 25, 26).

The MLR has been demonstrated to be an inflammatory marker (18, 27). Our study also suggested that the MLR was positively related to some inflammatory factors, such as PCT and CRP levels. In the study conducted by Hao-Ran Cheng, patients with stroke-associated pneumonia (SAP) had a higher MLR than non-SAP patients after AIS (28).

Previous studies have demonstrated that ischemia-reperfusion and inflammation play critical roles in the pathophysiology of AKI (18, 19). The MLR and NLR have been proposed as predictive factors for prognosis in patients diagnosed with ischemic stroke (28, 29). The NLR has been demonstrated to be a predictor of AKI in different populations (30, 31). In this study, we found that the MLR and the NLR were predictive of an increased rate of kidney damage in AHS patients. Therefore, the MLR and NLR may be easy, convenient, and cost-effective tools for the prediction of AKI in clinical practice.

In addition, we explored the association of the MLR and the NLR as well as that of AKI with in-hospital mortality and found that both the MLR and AKI were positively related to in-hospital mortality, with associated ORs of 2.09 and 2.95, respectively. However, in this study, in contrast to earlier studies, the NLR did not show an association with in-hospital mortality (29). The reason may be that the research endpoint was in-hospital mortality, not 3-month mortality. The population was patients with AHS in the NSICU, and most patients were critically ill. In the study, only 9 (0.9%) patients received RRT, and there were significant differences between patients with AKI and patients with no AKI. Although, in the study, most patients with AKI in stage 1 and stage 2 were patients who received timely treatment with good prognoses.

Some limitations should be acknowledged in the study. First, our investigation was a single-center retrospective observational study in which confounding factors and selective biases existed. Second, because diuretics can impact urine production and the patients were admitted to the NSICU, some patients may have experienced altered urine production and weight. Histological confirmation was not always performed in the clinic, and the diagnosis of AKI was based on the sCr level. As a result, the true prevalence of AKI may have been underestimated. Third, we only analyzed the MLR at the NSICU admission. We acknowledged that a dynamic measurement of the MLR and NLR could be more accurate for predicting AKI. Lastly, the study only looked at short-term prognoses, but future studies should focus on the long-term monitoring of subjects with AKI. Larger multicenter prospective studies are needed to confirm the value of such biomarkers in AHS-associated AKI.

## Conclusions

Our findings support that MLR and NLR measurements in patients diagnosed with AHS at admission could be valuable tools for identifying patients at high risk of early AKI. The MLR was also positively associated with in-hospital mortality and the NLR showed a weak ability to predict in-hospital mortality.

## Data Availability Statement

The original contributions presented in the study are included in the article/supplementary material, further inquiries can be directed to the corresponding author.

## Ethics Statement

Written informed consent was obtained from the individual(s) for the publication of any potentially identifiable images or data included in this article.

## Author Contributions

FJ and BY were involved in drafting the manuscript, conceptualization, design, involved in the interpretation of data, the critical revision of the manuscript for important intellectual content, and funding resource acquisitions. JL, XY, RZ, RL, and JR obtained all patient data. XL and SZ were involved in the data analysis. All authors gave their final approval for the submission.

## Funding

The work was supported by the Foundation of Hunan Provincial Health Commission Technology (202103050110 and 202103052372), Hunan Provincial Science Technology Foundation (2020JJ4550), and Hengyang City Guidance Plan Program (202121034630).

## Conflict of Interest

The authors declare that the research was conducted in the absence of any commercial or financial relationships that could be construed as a potential conflict of interest.

## Publisher's Note

All claims expressed in this article are solely those of the authors and do not necessarily represent those of their affiliated organizations, or those of the publisher, the editors and the reviewers. Any product that may be evaluated in this article, or claim that may be made by its manufacturer, is not guaranteed or endorsed by the publisher.
